# A Systematic Review of the Efficacy and Safety of Mulberry Formulations for Chemotherapy- and/or Radiotherapy-Induced Oral Mucositis

**DOI:** 10.7759/cureus.52340

**Published:** 2024-01-15

**Authors:** J Raghunand Sindhe, V Asha, Muthukrishnan Arvind, Shaik Shabana, A Sowbhagya Lakshmi, Khandekar Tanvi, Gimre Ananta

**Affiliations:** 1 Dentistry, Health Care Global Hospitals, Bangalore, IND; 2 Oral Medicine and Radiology, The Oxford Dental College and Hospital, Bangalore, IND; 3 Oral Medicine and Radiology, Saveetha Dental College and Hospitals, Chennai, IND; 4 Oral Medicine and Radiology, Al Ameen Dental College, Bijapur, IND; 5 Evidence Synthesis, coGuide Academy, Bangalore, IND

**Keywords:** quality of life, mulberry formulations, chemotherapy, radiotherapy, oral mucositis

## Abstract

Oral mucositis (OM) is one of the common side effects of radiotherapy and chemotherapy. It is an extremely painful condition characterized by erythema, edema, and ulceration of the oral mucosa. Many plant-based and chemical formulations are used to prevent OM. The aim of the study is to evaluate the efficacy and safety of different black mulberry formulations in chemotherapy and/or radiotherapy-induced OM.

A systematic search was performed using PubMed, Excerpta Medica database (Embase), the Cochrane Library, and Web of Science databases for articles published until March 2023. We have included studies conducted on people undergoing chemotherapy and/or radiotherapy and compared the effect of any mulberry formulation with other interventions. Out of 30 articles retrieved, four articles with a cumulative sample size of (N = 297) were included in the review. Mulberry formulations were compared with no intervention, grape molasses, chlorhexidine, and sodium bicarbonate. Out of the four articles, in three articles, mulberry formulations showed a significant decrease in grade 2 and grade 3 OM and also showed better prevention of OM as compared to the other intervention and control groups, and in one article, the grape molasses was more preventive for the occurrence of OM. Mulberry showed a significant decrease in dry mouth. Mulberry showed more improvement in the pain score and quality of life. The incidence and severity were lower in the mulberry group than in other interventions. One article showed less weight loss, and another article showed gradual weight gain from the use of mulberries. From this, we conclude that mulberry is effective for the treatment of OM. Mulberry also shows improvement in the pain score and quality of life.

## Introduction and background

Oral mucositis (OM) is a common side effect of radiation therapy (RT) to the head and neck, chemotherapy, chemoradiotherapy (CRT), and hematopoietic stem cell transplantation (HSCT) [1]. The reported incidence of OM ranges between 30% and 40% following chemotherapy for cancer and may increase to 60%-85% following HSCT and reach as high as nearly 90% following CRT for head and neck cancers (HNC) [2].

There are five stages of OM induced by radiotherapy and chemotherapy that occur sequentially, including initiation, signaling, amplification, ulceration, and healing [3]. Firstly, there is the initiation phase, in which cells get injured by radiotherapy and chemotherapy, which commonly occurs indirectly via reactive oxygen species or directly by damaging the DNA. This activates a series of enzyme and transcription factors, culminating in increased genetic expression and the production of multiple inflammatory cytokines like tumor necrosis factor-alpha (TNF-α), interleukin (IL)-1β, and IL-6. This will culminate in a vicious cycle of inflammation, tissue damage, ulceration, and microbial colonization. [4] Other than chemotherapy and/or radiotherapy, there are some other patient-related risk factors that can lead to OM, such as age, gender, smoking, and alcohol consumption. There are some disease-related risk factors, such as a history of cancer treatment, renal dysfunction, poor oral hygiene, undernutrition, dry mouth, etc., that may also predispose a person to OM [5].

Oral mucositis usually begins as grade I (erythema) by the second week, going through stages of grade II (focal areas of desquamation) by the third week, and grade III (confluent mucositis) by the fourth to fifth week following RT [6]. Further worsening leads to grade IV ulceration [7]. Severe OM (grade III or IV) may lead to local or systemic infectious complications, difficulty chewing and swallowing, loss of weight, malnutrition, treatment discontinuation, or even mortality in the worst cases [8]. Multiple objective measurement scales, like the World Health Organization (WHO) scale, the National Cancer Institute (NCI) Common Terminology Criteria for Adverse Events (CTCAE) version 3.0, and the Oral Mucositis Assessment Scale (OMAS), are used to assess OM [9].

In 2014, evidence-based clinical practice guidelines to manage OM were published by the Multinational Association of Supportive Care in Cancer and the International Society of Oral Oncology (MASCC/ISOO) [10]. As per the guidelines, standardized oral care, including brushing with a soft toothbrush, flossing, non-medicated oral rinsing, and chlorhexidine mouth rinse, was recommended. Also, some other therapeutic interventions are included, such as cryotherapy, growth factors, anti-inflammatory drugs such as benzydamine hydrochloride, and the use of low-level laser therapy [8].

Black mulberry molasses is a plant-based medication that is largely used as a traditional management method for OM. Mulberry extracts contain a high content of phenolics and flavonoids with anti-inflammatory and antioxidant properties [10]. Studies in patients with cancer have shown that black mulberry molasses prevents gingival sensitivity and dysphagia, slows down the formation of mucositis, and decreases the seriousness of mucositis. In the treatment of tonsillitis and the healing of oral and dental wounds, black mulberry is particularly effective because it contains papiriflavonal A, kuraridin, saforaflavanon D, and saforaiso flavanon A, which give it good antifungal and strong antimicrobial activity. With all this, 2-arylbenzofurans is also present in black mulberry, has an antimicrobial effect on methicillin-resistant *staphylococci*, and also has a strong antioxidant effect [11]. Though there are multiple studies conducted on the effectiveness of mulberry formulations to prevent or treat chemotherapy and/or radiotherapy-induced OM, there is no systematic review or meta-analysis on the subject synthesizing the available evidence. Hence, the purpose of this systematic review is to evaluate the efficacy and safety of black mulberry in chemotherapy and/or radiotherapy-induced OM.

## Review

Study design

The current systematic review was conducted as per Preferred Reporting Items for Systematic Reviews and Meta-Analyses (PRISMA) guidelines.

Eligibility criteria

The review included studies investigating the effects of different mulberry formulations to manage chemotherapy and/or radiotherapy-induced OM among cancer patients. The reviewed studies included randomized controlled clinical trials (RCTs), non-randomized controlled trials (NRCTs), and analytical cohort studies, all published in English. The review excluded studies on patients having OM due to chronic obstructive pulmonary disease (COPD) and other non-malignant conditions, case studies, and review articles.

Information sources

We systematically searched PubMed, the Excerpta Medica database (Embase), Web of Science, the Cochrane Library, and clinicaltrial.gov until April 2023.

Search strategy

The search was conducted using subject headings corresponding to particular databases (medical subject headings (MeSH) in PubMed and Emtree in Embase), alternate terms, and supplementary concepts. The MeSh terms included “mulberry” and “oral mucositis." We listed out keywords and their synonyms and conducted free text searching. Suitable search functions like truncations, wildcards, and proximity searching were used. Then, we combined the individual search results with suitable Boolean operators. We also screened the reference lists of selected articles using a simple Google search (Google Inc, Mountainview, CA) so as not to miss any articles not retrieved in the database search.

Selection process

Search output from all the individual databases was imported to a specific Zotero library (Corporation for Digital Scholarship, Vienna, VA), and duplicates were removed by the automatic duplicate identification feature and merging. The final list, after removing duplicates, was exported to Rayyan software (Rayyan Systems Inc., Cambridge, MA).

**Figure 1:**
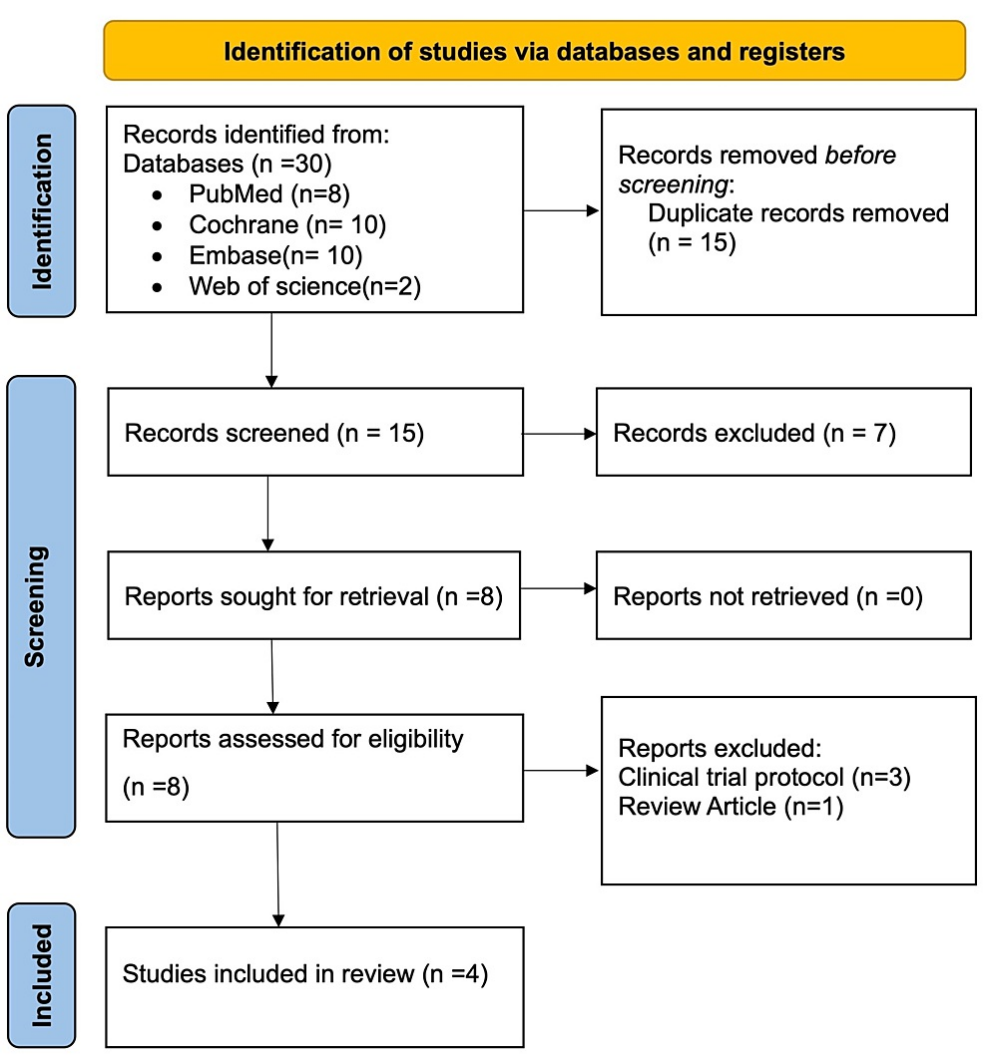
PRISMA flowchart PRISMA: Preferred Reporting Items for Systematic Reviews and Meta-Analyses

Further duplicate removal was done using the automatic duplicate detection function, Rayyan, and quick manual screening of the bibliographic details. The final list, after removing duplicates, was taken up for level 1 screening. Two researchers carefully screened the titles and abstracts and shortlisted studies that were likely to satisfy the inclusion criteria. Disagreements between the two reviewers were resolved by involving a third reviewer. Full-text articles for all the shortlisted studies were retrieved for level 2 screening. Full-text articles were read thoroughly by two independent reviewers blinded to each other, and a decision was marked in Rayyan. Conflicts were resolved in the same manner as level 1 screening. The results of the screening and selection process are documented in a PRISMA flowchart (Figure 1).

Data collection process

Comprehensive data extraction was done by two independent reviewers from eligible studies to evaluate the efficacy and safety of mulberry formulations among patients with OM due to radiotherapy and/or chemotherapy. Data were extracted on a data extraction form. Attempts were made to obtain all the relevant missing data by contacting the primary authors. A double review of the extracted data was performed to minimize data extraction errors and missing data.

Data items

The data extraction template contained bibliometric parameters like author name, year of publication, country of the study, characteristics of the study population, details of the intervention and comparator groups, sample size, and outcomes like OM, quality of life, body weight, pain score, etc.

Synthesis methods

The included studies were assessed for the presence of clinical and methodological heterogeneity. The study population, study design, details of the intervention, comparator interventions, and outcome measures were significantly heterogeneous across the included studies. Hence, the results were presented through qualitative synthesis, and no meta-analysis was performed.

Study of risk of bias assessment

Risk of bias assessment was done using the Cochrane Risk-of-Bias Tool for Randomized Trials (RoB 2) for RCTs and the Risk of Bias in Non-randomised Studies - of Interventions (ROBINS-I) for non-randomized studies of interventions. Assessment was done as recommended in the Cochrane Handbook of Systematic Reviews [12, 13].

Results

Study Selection

From all the sources, a total of 30 articles were retrieved, of which 15 were duplicates. Titles and abstracts of 15 articles screened at levels 1 and 7 were excluded. Eight studies were shortlisted for full-text retrieval and screening. Following the full-text screening, four studies with a pooled sample size of 297 were included in the final qualitative synthesis. Among the four excluded studies, one was because of the wrong study design, and three studies were excluded because they followed clinical trial protocols.

Study Characteristics

Out of the four studies included, one was a prospective study, one was a randomized pilot study, one was an NRCT, and one was an RCT. All the studies were conducted in Turkey. The study population in the prospective study was patients with HNC. All patients were treated with intensity-modulated radiation therapy (IMRT) and CRT [10]. In a randomized pilot study, cancer patients receiving chemotherapy for hematological malignancies, breast cancer, and lung cancer were included [14]. The NRCT included patients undergoing stem cell transplantation [15]. The RCT included patients with HNC receiving RT for oropharyngeal mucosa [16]. Oral mucositis assessment was the common outcome reported by all four studies. Pain, quality of life, weight assessment, and dry mouth were the other outcomes reported by specific studies. The incidence and severity of OM were reported by one study (Table 1).

**Table 1:** Characteristics of included studies IMRT: intensity-modulated radiation therapy; C1: comparator group 1; C2: comparator group 2

S. No.	Author name and year	Country	Study design	Study population	Intervention	Comparators	Outcomes
1	Yuce Sari et al., 2022 [10]	Turkey	Prospective study	Head and neck cancer treated with IMRT and chemotherapy for specific cancers.	Black mulberry molasses	C1: Grape molasses gargle and C2: Control	Oral mucositis, quality of life changes, pain, and weight change
2	Karabey et al., 2022 [14]	Turkey	Randomized pilot study	Undergoing chemotherapy for hematological malignancies, breast, and lung cancer.	Black mulberry extract	Sodium bicarbonate	Oral mucositis, weight change, and severity of dry mouth
3	Harman et al., 2019 [15]	Turkey	Non- randomized controlled trial	Stem cell transplantation	Black mulberry syrup	C1: Calcium and phosphate solution and C2: chlorhexidine gluconate and benzydamine hydrochloride solution.	Oral mucositis
4	Demir et al., 2017 [16]	Turkey	Randomized controlled trial	Head and neck cancer patients receiving radiation therapy for oropharyngeal mucosa	Radiation therapy plus mulberry molasses	Radiation therapy alone	Oral mucositis, pain score, incidence and severity of oral mucositis, quality of life changes

Description of Interventions​​​​​​​

Black mulberry extracts were used in different formulations like molasses, syrup, and extracts as gargles. All the studies have used mulberry extracts as oral gargles. The frequency varied from twice daily to four times daily. Comparator groups included no intervention, calcium and phosphate solutions, chlorhexidine gluconate and benzydamine hydrochloride solutions, grape molasses gargle, and sodium bicarbonate solutions (Table 1).

Outcomes

Oral Mucositis Assessment

Oral mucositis was the primary outcome in all four articles.

As per the study by Yuce Sari et al., by the sixth week, the proportion of patients with grade 2 OM was 27%, 19%, and 36% of those taking black mulberry molasses, grape molasses, and the control group, respectively. By the sixth week, 32%, 23%, and 21% of patients taking black mulberry molasses, grape molasses, and the control group, respectively, were affected by grade 3 OM [10].

In the study by Demir Doğan et al., the proportion of participants affected by grade 2 OM was 63.9% and 53.7% among the mulberry molasses and control groups by the fifth week. Grade 3 OM was reported in 2.7% of patients in the mulberry group and 26.8% of patients in the control group by the fifth week. The proportion of both grade 2 and grade 3 OM gradually decreased in both groups by the seventh week. Grade 2 OM was reported in 2.8% and 9.8% of groups with and without mulberry extract, respectively. No patient taking mulberry molasses had grade 3 OM, whereas 2.4% of patients taking only radiotherapy had it. By the 90th day, all the patients were cured of OM [16].

In the article by Karabey et al., the highest mean score of the oral assessment guide was 1.92 and 2.06 in patients taking black mulberry extract and sodium bicarbonate, respectively. By the third week, the mean score had reduced to 1.21 and 1.77 in the black mulberry extract and sodium bicarbonate groups, respectively [14].

In a study by Harman et al., by the third week, 8%, 7.1%, and 20% of patients taking black mulberry syrup, calcium and phosphate solutions, and chlorhexidine gluconate and benzydamine hydrochloride solutions, respectively, were affected by grade 2 OM. Grade 3 OM was reported in 8%, 7.1%, and 13.3% of patients taking black mulberry syrup, calcium and phosphate solutions, and chlorhexidine, gluconate, and benzydamine hydrochloride solutions. They were affected by grade 3 OM by the third week [15] (Table 2).

**Table 2:** Assessment of oral mucositis * not applicable

Author	Intervention	Sample size	Grade	Week 1	Week 2	Week 3	Week 4	Week 5	Week 6	Week 7
Yuce Sari et al., 2022 [10]	Black mulberry molasses	40	Grade 2	0	*	8 (20%)	*	*	10 (27%)	*
			Grade 3	1(2%)	*	1 (2%)	*	*	13 (32%)	*
	Grape molasses	40	Grade 2	1(2%)	*	8 (20%)	*	*	8 (20%)	*
			Grade 3	1(2%)	*	2 (5%)	*	*	9 (23%)	*
	Control	14	Grade 2	0	*	5 (36%)	*	*	5 (36%)	*
			Grade 3	0	*	0	*	*	3 (21%)	*
Karabey et al., 2022 [14]	Black mulberry extract	20	Mean	1.92	1.53	1.21	*	*	*	*
	Sodium bicarbonate	20	Mean	2.06	1.9	1.77	*	*	*	*
Harman et al., 2019 [15]	Black mulberry syrup	25	Grade 2	0	2 (8%)	2 (8%)	*	*	*	*
			Grade 3	0	0	2 (8%)	*	*	*	*
	Calcium and phosphate solution	28	Grade 2	0	5 (17.9%)	2 (7.1%)	*	*	*	*
			Grade 3	0	0	2 (7.1%)	*	*	*	*
	Chlorhexidine gluconate and benzydamine hydrochloride solution	30	Grade 2	1 (3.3%)	5 (16.7%)	6 (20%)	*	*	*	*
			Grade 3	0	2 (6.7%)	4 (13.3%)	*	*	*	*
Demir Doğan et al., 2017 [16]	Radiation therapy plus mulberry molasses	38	Grade 2	0	0	0	2(5.6%)	23 (63.9%)	6 (16.7%)	1 (2.8%)
			Grade 3	0	0	1 (2.7%)	0	1 (2.7%)	1 (2.7%)	0
	Radiation therapy	42	Grade 2	0	1 (2.4%)	1 (2.4%)	8 (19.5%)	22 (53.7%)	13 (31.7%)	4 (9.8%)
			Grade 3	0	0	2 (4.9%)	3 (7.3%)	11 (26.8%)	7 (17.1%)	1 (2.4%)

Weight Assessment​​​​​​​

In one article by Yuce Sari et al., the mean body weight by the end of the first week was 72.55 kg, 72.87 kg, and 70.75 kg in patients taking black mulberry molasses, grape molasses, and the control group, respectively. By the sixth week, it had decreased to 69.22 kg, 62.3 kg, and 67.75 kg in patients with black mulberry molasses, grape molasses, and the control group, respectively [10]. In the study by Karabey et al., weight gain in the patients taking mulberry extract was significantly higher, and no significant difference was found in weight among the patients taking sodium bicarbonate [14] (Table 3).

**Table 3:** Assessment of weight, dry mouth, and pain RTOG: Radiation Therapy Oncology Group; EORTC: European Organization for Research and Treatment of Cancer

Author	Intervention	Sample size	Week 1	Week 2	Week 3	Week 6
Weight assessment						
Yuce Sari et al., 2022 [10]	Black mulberry molasses	40	72.55±11.75	Not applicable	71.68±11.32	69.22±11.3
	Grape molasses	40	72.87±17.43	Not applicable	68.02±19.9	62.3±19.9
	Control	14	70.75±13.38	Not applicable	69.42±10.69	67.75±9.93
Karabey et al., 2022 [14]	Black mulberry extract	20	65±8.97	65.3±8.79	66.7±7.98	Not applicable
	Sodium bicarbonate	20	68.55±12.03	68.47±12.54	68.76±12.51	Not applicable
Dry mouth (RTOG/EORTC) assessment						
Karabey et al., 2022 [14]	Black mulberry extract	20	2.18±0.5	1.31±0.71	0.54±0.03	Not applicable
	Sodium bicarbonate	20	2.45±0.75	2.05±0.51	1.55±0.51	Not applicable
Pain assessment						
Yuce Sari et al., 2022 (median (min–max)) [10]	Black mulberry molasses	40	0 (0–7)		2 (0–8)	3 (0–9)
	Grape molasses	40	0 (0–5)		1 (0–6)	0 (0–7)
	Control	14	0 (0–8)		4 (0–7)	5 (0–8)
Demir Doğan et al., 2017 (Overall mean) [16]	Radiation therapy plus mulberry molasses	38	4.44%			
	Radiation therapy	42	3.92%			

Dry Mouth Assessment​​​​​​​

In Karabey et al., the Radiation Therapy Oncology Group (RTOG) and the European Organization for Research and Treatment of Cancer (EORTC) scale were used for the assessment of dry mouth. The mean score in the first week was 2.18 and 2.45 in patients taking black mulberry extract and sodium bicarbonate, respectively. Then it decreased by the third week to 0.54 and 1.55 in the black mulberry extract and sodium bicarbonate groups, respectively [14] (Table 3).

Pain Assessment​​​​​​​

In the article by Demir Doğan et al., the average pain assessment score due to oral mucosa was lower in the patients taking mulberry molasses with radiotherapy than in the patients undergoing only radiotherapy at weekly follow-ups during treatment. Pain in the oral mucosa developed earlier in the control group as compared to the mulberry molasses group (3.92% vs. 4.44%, p = 0.005), which was statistically significant [16]. In the article by Yuce Sari et al., the median visual analog scale (VAS) score was used for the assessment of pain, which was three, 0, and five in patients taking black mulberry molasses, grape molasses, and the control group, respectively, by the sixth week [10] (Table 3).

Quality of Life Assessment

In the article by Yuce Sari et al., the EORTC-Head and Neck Cancer Module (HN35) module and the EORTC Quality of Life Questionnaire (QLQ-C30) scale were used for the assessment of quality of life. The EORTC HN35 scoring was higher, except for nutritional supplements, feeding tubes, and weight gain, in the control group than in the black mulberry molasses and grape molasses groups. The global health score was highest in the black mulberry molasses group [10]. In the article by Demir Dogan et al., the quality of life score was higher in patients taking only radiotherapy than in patients taking mulberry molasses with radiotherapy [16] (Table 4).

**Table 4:** Assessment of quality of life and incidence and severity EORTC HN35: European Organization for the Research and Treatment of Cancer-Head and Neck Cancer Module

Quality of life		
Author	Intervention	Outcome
Yuce Sari et al., 2022 [10]	Black mulberry molasses	EORTC HN35 scoring was higher in the control group than in the other two groups. The global health score was highest in the black mulberry molasses group
	Grape molasses	
	Control	
Demir Doğan et al., 2017 [16]	Radiation therapy plus mulberry molasses	Lower score
	Radiation therapy	Higher score
Incidence and severity		
Demir Doğan et al., 2017 [16]	Radiation therapy plus mulberry molasses	Higher score
	Radiation therapy	Lower score

Incidence and Severity Assessment​​​​​​​

In the article by Demir Doğan et al., the incidence and severity of oral mucositis were higher in patients undergoing radiotherapy than in patients taking black mulberry molasses with radiotherapy [16] (Table 4).

Risk of Bias Among Included Studies​​​​​​​

The risk of bias assessment showed that three of the four included studies had a high or serious risk of bias, primarily arising from poor randomization processes in one RCT and poor methodology of participant selection [10,14,15]. Only one RCT had a robust methodology with a low risk of bias [16] (Table 5).

**Table 5:** Overall risk of bias assessment

Study ID	Study design	Randomization process	Bias due to confounding	Bias in selection	Bias in the classification of interventions	Bias due to deviation from intervention	Bias due to missing data	Bias in outcome measurement	Selective reporting	Overall
Yuce Sari et al., 2022 [10]	Prospective study	Not applicable	Low	Serious	Low	Low	Low	Moderate	Low	Serious
Karabey et al., 2022 [14]	Randomized pilot study	High	Not applicable	Not applicable	Not applicable	Low	Low	Some concerns	Low	High
Harman et al., 2019 [15]	Non-randomized controlled trial	Not applicable	Low	Serious	Low	Low	Low	Moderate	Low	Serious
Demir Doğan et al., 2017 [16]	Randomized controlled trial	Low	Not applicable	Not applicable	Not applicable	Low	Low	Low	Low	Low

Discussion

The current systematic review is an attempt to synthesize the available evidence on the relative efficacy and safety of different formulations of mulberry as compared to other interventions in the prevention and management of OM. Oral mucositis assessment, weight changes, dry mouth severity, pain scale, and quality of life were the key outcomes considered.

In three of the four included articles, there was a significant reduction in the incidence of grade 2 and grade 3 oral mucositis by the use of mulberry formulations as compared to no intervention or other specified interventions. In one study, grape molasses showed slightly higher efficacy in preventing OM than black mulberry molasses. Weight loss was less with mulberry molasses as compared to grape molasses. The use of black mulberry extract showed more weight gain than the use of sodium bicarbonate. Dry mouth was significantly lessened with the use of black mulberry. The severity of pain was lower with mulberry molasses as compared to the control group. Grape molasses showed a lower severity of pain as compared to black mulberry molasses. The quality of life was also reported to be relatively higher with mulberry molasses.

Black mulberry formulations have been used for the prevention and treatment of oral mucositis in conditions other than cancer patients undergoing RT and/or CT. Considering the relative scarcity of studies, it might be useful to critically appraise the evidence from the current study in the context of wider evidence in other disease conditions. An RCT by Korkut et al. [11] on COPD patients showed oral care with black mulberry syrup accelerates mucositis healing and alleviates mucositis-related symptoms.

Apart from mulberry formulations, a wide range of plant-based formulations have been studied for their efficacy on oral mucositis. These plant-based anti-inflammatory agents included curcumin from *Curcuma longa*, acemannan from aloe vera, and licorice root from *Glycyrrhiza glabra* [17]. There are multiple systematic reviews of these agents. One review reported a reduced grade of mucositis, pain, erythema intensity, and ulcerative area with turmeric or curcumin [18]. Propolis mouthwash [19], oral cryotherapy [20], recombinant human KGF-1 (palifermin) [21], rebamipide mouthwash [22], use of probiotics [23], etc. were other different interventions evaluated for their efficacy and safety. Interventions based on local treatments with non-chemical methods like a prophylactic low-level laser (LLLT) and therapeutic LLLT [24] and photobiomodulation [25] were also documented to be effective. However, there are no head-to-head comparison studies assessing the relative superiority of these products as compared to mulberry formulations.

Based on the overall analysis of available scientific evidence for the prevention and management of OM, there are a plethora of interventions based on plant-based formulations, chemical substances, and physical methods. The overall direction of the evidence shows the relative superiority of many of these interventions as compared to no intervention. However, the evidence assessing the relative superiority of these interventions is quite patchy and diverse. From the clinical practitioner’s perspective, having a diverse range of interventions to choose from might be good. However, it may often leave them with a clinical dilemma about choosing the best intervention from the available list of interventions. Hence, there is a strong need to channel the research on the subject in a direction to establish the relative superiority of the available interventions by large-scale head-to-head comparison studies. Also, exploring the dimensions of cost-effectiveness, acceptability, and accessibility of the interventions in different settings might be of strong practice relevance. Possible differences in effectiveness in different population subgroups based on the type of primary disease, comorbidities, etc. is also an important dimension to explore.

The current review is the first attempt to synthesize the available evidence of various mulberry formulations in the prevention and/or treatment of OM among cancer patients. A comprehensive search of major databases, methodological adherence to PRISMA guidelines, and a detailed presentation of multiple outcomes related to oral mucositis and its impact on weight and quality of life are the strengths of the current review.

Significant methodological heterogeneity, precluding quantitative synthesis, is the key limitation. All the studies published were from Turkey; hence, the generalizability of the study findings is very limited. The overall quality and trustworthiness of the summary conclusions of this review are low due to the high risk of bias. Primary sources of serious bias were participant selection, the non-description of the randomization process, and allocation concealment. Lack of blinding in RCTs was another source of possible bias in outcome assessment. Studies have not really made efforts to quantify the level of adherence to the intervention, raising some concerns about bias and various levels of deviation from interventions. This clearly indicates the need for well-designed, scientifically robust RCTs on the subject.

## Conclusions

Mulberry formulations have been compared with no intervention and a wide variety of other chemical formulations. Except for grape molasses, mulberry formulations have demonstrated superior efficacy in preventing grade 2 and 3 OM. Mulberry formulations also demonstrated superior effects, reducing pain due to OM, minimizing weight loss, promoting weight gain, and improving quality of life. No clear practice recommendations can be made from the current review, considering the diversity of comparison groups and the poor quality of the available evidence. Scientifically robust RCTs with head-to-head comparisons of different plant-based formulations are needed to establish relative superiority, acceptability, and cost-effectiveness to guide clinical decisions.
